# Asymmetric Ensemble of Asymmetric U-Net Models for Brain Tumor Segmentation With Uncertainty Estimation

**DOI:** 10.3389/fneur.2021.609646

**Published:** 2021-09-30

**Authors:** Sarahi Rosas-Gonzalez, Taibou Birgui-Sekou, Moncef Hidane, Ilyess Zemmoura, Clovis Tauber

**Affiliations:** ^1^UMR Inserm U1253, iBrain, Université de Tours, Inserm, Tours, France; ^2^LIFAT EA 6300, INSA Centre Val de Loire, Université de Tours, Tours, France

**Keywords:** brain tumor segmentation, deep-learning, BraTS, multi-input, multi- view, inception, uncertainties, 2.5D convolutions

## Abstract

Accurate brain tumor segmentation is crucial for clinical assessment, follow-up, and subsequent treatment of gliomas. While convolutional neural networks (CNN) have become state of the art in this task, most proposed models either use 2D architectures ignoring 3D contextual information or 3D models requiring large memory capacity and extensive learning databases. In this study, an ensemble of two kinds of U-Net-like models based on both 3D and 2.5D convolutions is proposed to segment multimodal magnetic resonance images (MRI). The 3D model uses concatenated data in a modified U-Net architecture. In contrast, the 2.5D model is based on a multi-input strategy to extract low-level features from each modality independently and on a new 2.5D Multi-View Inception block that aims to merge features from different views of a 3D image aggregating multi-scale features. The Asymmetric Ensemble of Asymmetric U-Net (AE AU-Net) based on both is designed to find a balance between increasing multi-scale and 3D contextual information extraction and keeping memory consumption low. Experiments on 2019 dataset show that our model improves enhancing tumor sub-region segmentation. Overall, performance is comparable with state-of-the-art results, although with less learning data or memory requirements. In addition, we provide voxel-wise and structure-wise uncertainties of the segmentation results, and we have established qualitative and quantitative relationships between uncertainty and prediction errors. Dice similarity coefficient for the whole tumor, tumor core, and tumor enhancing regions on BraTS 2019 validation dataset were 0.902, 0.815, and 0.773. We also applied our method in BraTS 2018 with corresponding Dice score values of 0.908, 0.838, and 0.800.

## Introduction

Glioma is the most frequent primary brain tumor (1). It has its origin in glial cells and can be classified into I to IV grades, depending on phenotypic cell characteristics. In this grading system, low-grade gliomas (LGGs) correspond to grades I and II, whereas high-grade gliomas (HGGs) are grades III and IV. The primary treatment is surgical resection followed by radiation therapy and/or chemotherapy.

MRI is a non-invasive imaging technique commonly used for diagnosis, surgery planning, and follow-up of brain tumors due to its high resolution on brain structures. Currently, tumor regions are segmented manually from MRI images by radiologists, but due to the high variability in image appearance, the process is very time consuming and challenging, and inter-observer reproducibility is considerably low (2). Since accurate tumor segmentation is determinant for surgery, follow-up, and subsequent treatment of glioma, finding an automatic and reproducible solution may save time for physicians and contribute to improving the clinical assessment of glioma patients. Based on this observation, the Multimodal Brain Tumor Segmentation Challenge (BraTS) aims at stimulating the development and the comparison of the state-of-the-art segmentation algorithms by making available an extensive pre-operative multimodal MRI dataset provided with ground truth labels for three tumor tissues: enhancing tumor, the peritumoral edema, and the necrotic and non-enhancing tumor core. This dataset contains four modalities: T2-weighted (T2), fluid-attenuated inversion recovery (FLAIR), T1-weighted (T1), and T1 with contrast-enhancing gadolinium (T1c) (3–7).

Modern convolutional neural networks (CNNs) are currently state-of-the-art in many medical image analysis applications, including brain tumor segmentation (8). CNNs are hierarchical groups within filter banks that extract increasingly high-level image features by feeding the output of each layer to the next one. Recently, Ronneberger et al. (9) proposed an effective U-Net model, a fully convolutional network (FCN) encoder/decoder architecture. The encoder module consists of multiple connected convolution layers that aim to gradually reduce the spatial dimension of feature maps and capture high-level semantic features appropriate for class discrimination. The decoder module uses upsampling layers to recover the spatial extent and object representation. The main contribution of U-Net is that, while upsampling and going deeper into the network, the model concatenates the higher resolution features from the encoder path with the upsampled features in the asymmetric decoder path to better localize and learn representations in following convolutions. The U-Net architecture is one of the most widely used for brain tumor segmentation, and its versatile and straightforward architecture has been successfully used in numerous segmentation tasks (10–14). All top-performing participants in the last two editions of the BraTS challenge used this architecture (15–22).

While 3D CNN can provide global context information of volumetric tumors, the large size of the images makes the use of 3D convolutions very memory demanding, which limits the patches and batch size, as well as the number of layers and filters that can be used (23). Consequently, the use of 2D convolutions for slice-by-slice segmentation is also a common practice that reduces memory requirement (24). Multi-view approaches have also been developed to address the same problem. McKinley et al. (18) and Xue et al. (25) showed that applying 2D networks in axial, sagittal, and coronal views and combining their results can recover 3D spatial information. Recently, one of the top-performing submissions in the BraTS 2019 challenge (20) proposed a hybrid model that goes from 3D to 2D convolutions extracting two-dimensional features in each of the orthogonal planes and then combines the results in an ensemble model. Wang et al. (16) demonstrated that using three 2.5D networks to obtain separate predictions from three orthogonal views and fusing them at test time can provide more accurate segmentations than using an equivalent 3D isotropic network. While they require the training and optimization of several models, ensemble models are currently the top-performing methods for brain tumor segmentation.

On the other hand, some strategies have been implemented to aggregate multi-scale features. Cahall et al. (26) showed a significant improvement in brain tumor segmentation by incorporating Inception blocks (27) into a 2D U-Net architecture. Wang et al. (16) and McKinley et al. (20) used dilated convolutions (28) in their architecture with the same aim of obtaining both local and more global features. While significant, aggregating multi-scale features is limited by the requirement of more memory capacity. To address this, the use of Inception modules has been incorporated into 2D networks (26), not taking advantage of 3D contextual information. In addition, Inception modules have been integrated into a cascade network approach (29). The model first learns the whole tumor, then the tumor core, and finally the enhancing tumor region. This method requires three different networks and thus increases the training and inference time. Another approach to extract multi-scale features uses dilated convolutions. This operation was explicitly designed for semantic segmentation and tackled the dilemma of obtaining multi-scale aggregation without losing full resolution, increasing the receptive field (28). Wang et al. (16) and McKinley et al. (20) implemented different dilation rates in sequential convolutions; nevertheless, it has not been used to extract multi-scale features in a parallel way in a single layer and, if not applied carefully, can cause gridding effects, especially in small regions (30).

In terms of accuracy and precision, the performance of CNNs are currently comparable with human-level performance or even better in many medical image analysis applications (31). However, CNNs have also often been shown to produce inaccurate and unreliable probability estimates (32, 33). This has drawn attention to the importance of uncertainty estimation in CNN (34). Among other advantages, the measurement of uncertainties would enable knowing how confident a method is in implementing a particular task. This information can facilitate CNN's incorporation into clinical practice and serve the end-user by focusing attention on areas with high uncertainty (35).

In this study, we propose an approach that addresses a current challenge of brain tumor segmentation, keeping reduced memory requirements while benefiting from multi-scale 3D information. To do so, we propose an ensemble model, called Asymmetric Ensemble Asymmetric U-Net (AE AU-Net), based on an Asymmetrical 3D residual U-Net (AU-Net) using two different kinds of inputs: (1) concatenated multimodal 3D MRI data (3D AU-Net) and (2) a 2.5D Multi-View Inception Multi-Input module (2.5D AU-Net). The proposed AU-Net is wider in the encoding path to extract more semantic features, has residual blocks in each level to increase training speed, and additive skip connections between the encoding and decoding path instead of a concatenation operation to reduce the memory consumption. The proposed 2.5D strategy allows us to extract low-level features from each modality independently. In this way, the model can retrieve specific details related to tumor appearance from the most informative modalities, without the risk of being lost when combined during the downsampling. The Multi-View Inception block aims to merge features from both different views and different scales simultaneously, seeking a balance between 3D information usage and memory footprint. In addition, we use an ensemble of models to improve our segmentation results and the generalization power of our method and also as a way to measure epistemic uncertainty and estimate structure-wise uncertainty.

## Methods

### Network Architecture

**Figure 1:**
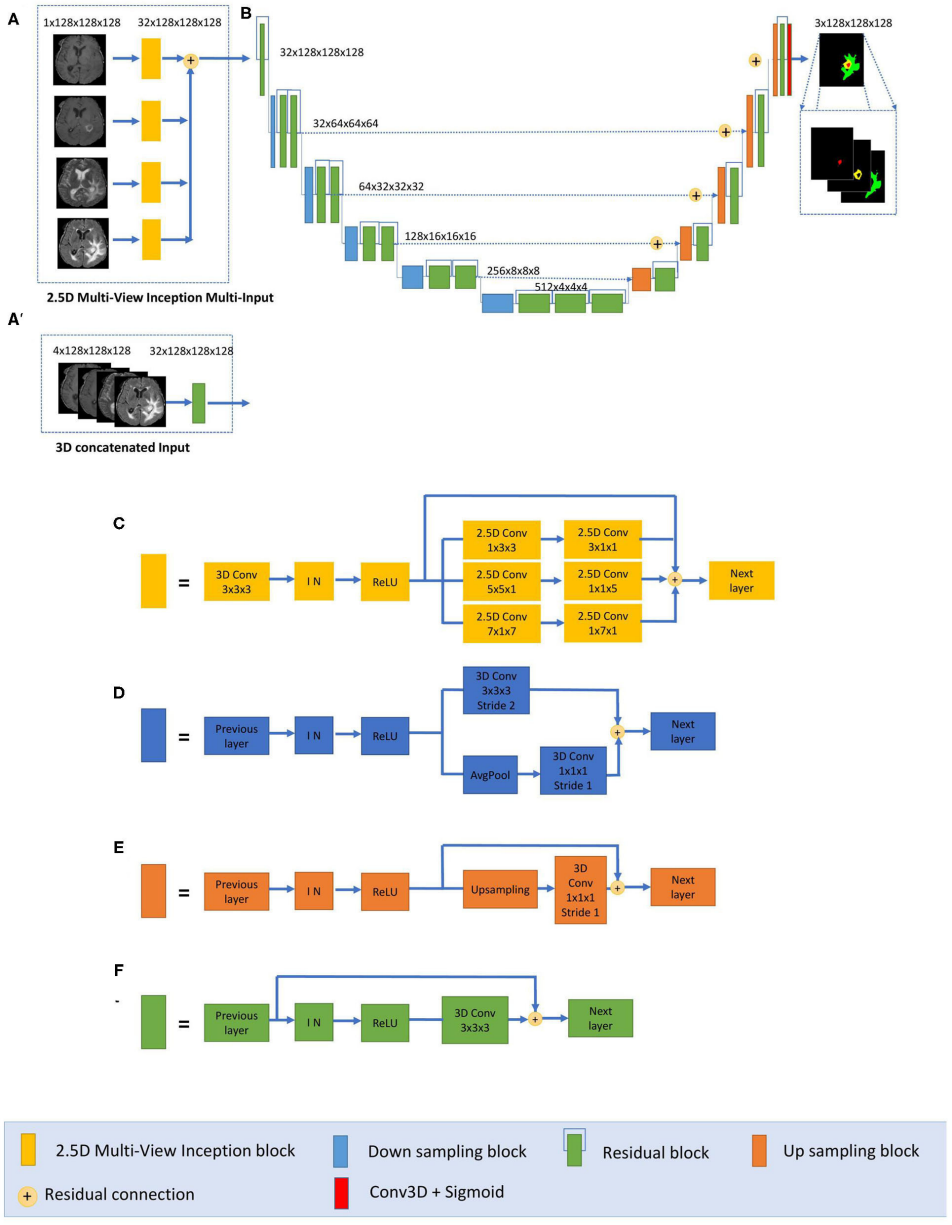
Proposed 2.5D Asymmetric U-Net and 3D Asymmetric U-Net. **(A)** 2.5D Multi-View Inception Multi-input. **(A′)** 3D concatenated input. **(B)** An asymmetric 3D U-Net architecture using residual blocks, instance normalization (IN), and additive skip connections between encoding and decoding sections. **(C)** 2.5D Multi-View Inception block. **(D)** Down sampling block. **(E)** Up sampling block. **(F)** Residual block.

We have developed a modified U-Net architecture with five-level asymmetric descending and ascending parts implemented with two kinds of inputs for ensemble modeling (Figure 1). While the first input is a classical concatenation of 3D MRI data, the second one is a novel 2.5D Multi-View Inception Multi-Input module aiming to extract low-level textural features of the tumor. Details of the modified U-Net proposed inputs and assembling strategy are provided.

#### Asymmetric U-Net (AU-Net)

The main component of our network is a modified 3D U-Net architecture of five levels (Figure 1B). Traditional 3D U-Net architecture has a symmetrical decoder and encoder paths. The first path is associated with the extraction of semantic information to make local predictions, while the second section is related to the recovery of the global structure. AU-Net is wider in the encoding path than in the decoding path, to extract more semantic features while keeping memory usage lower. We added twice more convolutional blocks in the encoding path than in the decoding section to achieve this. The standard U-Net architecture also does not get enough semantic information in the downsampling path because of the limited receptive fields. We added parallel paths with convolutions with two different filter sizes in the downsampling blocks (Figure 1D).

This architecture uses residual blocks (Figure 1F) instead of a simple sequence of convolutions. The residual blocks (36) are obtained by a short skip connection and element-wise addition operation between each block's input and output feature maps. This simple algorithm does not add additional training parameters to the network, and it has been shown to be beneficial for faster convergence, reducing training time (37). We also used additive skip connections between the encoding outputs and decoding feature maps of equivalent size instead of using a concatenation operation. This enables reducing memory consumption while maintaining similar performances compared with using concatenation operations. Finally, each level of the encoding pathway consists of two residual blocks (green blocks in Figure 1) followed by a downsampling block (blue blocks). We used convolutions with stride equal to 2 for downsizing image dimension, maintaining the same number of filters in the first two levels and then increasing the number of features in each level with an initial number of filters equal to 32. Thus, the spatial dimension of the initial training patches of size 128 × 128 × 128 was reduced by a factor of 32 in the encoding path. In the decoding path, we used only one residual block in each level, and we used upsampling layers, which duplicate values in a sliding window to increase the dimension. The upsampling layers are directly followed by convolution ones. This technique of upsampling helps to avoid the checkerboard pattern generated by transposed convolution (deconvolution) layers as experimented by Odena et al. (38).

The final activation layer uses a sigmoid as an activation function to output three prediction maps of the same size as the initial training patches into three channels corresponding to the regions: whole tumor, tumor core, and enhancing tumor. We chose to classify the three regions by doing three binary classifications, one for each region.

#### 3D AU-Net

The proposed AU-Net was used with two different kinds of inputs. In the first one, called 3D AU-Net, 3D concatenated MRI sequences are used as the input to capture classical multimodal information from the different MRI sequences. This input is presented in Figure 1A′.

#### 2.5D AU-Net

The second type of input is proposed to capture the textural and multi-scale features of the tumor better. This second input is a 2.5D input for the AU-Net, with a different strategy. A multi-input module (Figure 1A) has been developed to maximize learning from independent features associated with each imaging modality before merging into the encoding/decoding architecture, thus avoiding the loss of specific information provided by each modality. While most previous architectures concatenate all the MRI modalities as a single multi-channel input, we propose to extract low-level features associated with tissue appearance from each modality independently. To do so, we divide the input into four paths, one for each imaging modality, to extract features from each modality independently, and then we merge them as an input of the proposed AU-Net.

In addition, we propose that each of these four paths contains what we define as a 2.5D Multi-View Inception module (Figure 1C) that allows the extraction of features in the different orthogonal views of the 3D image: axial, coronal, and sagittal planes and different scales, merging them all in each forward pass.

**Figure 2:**
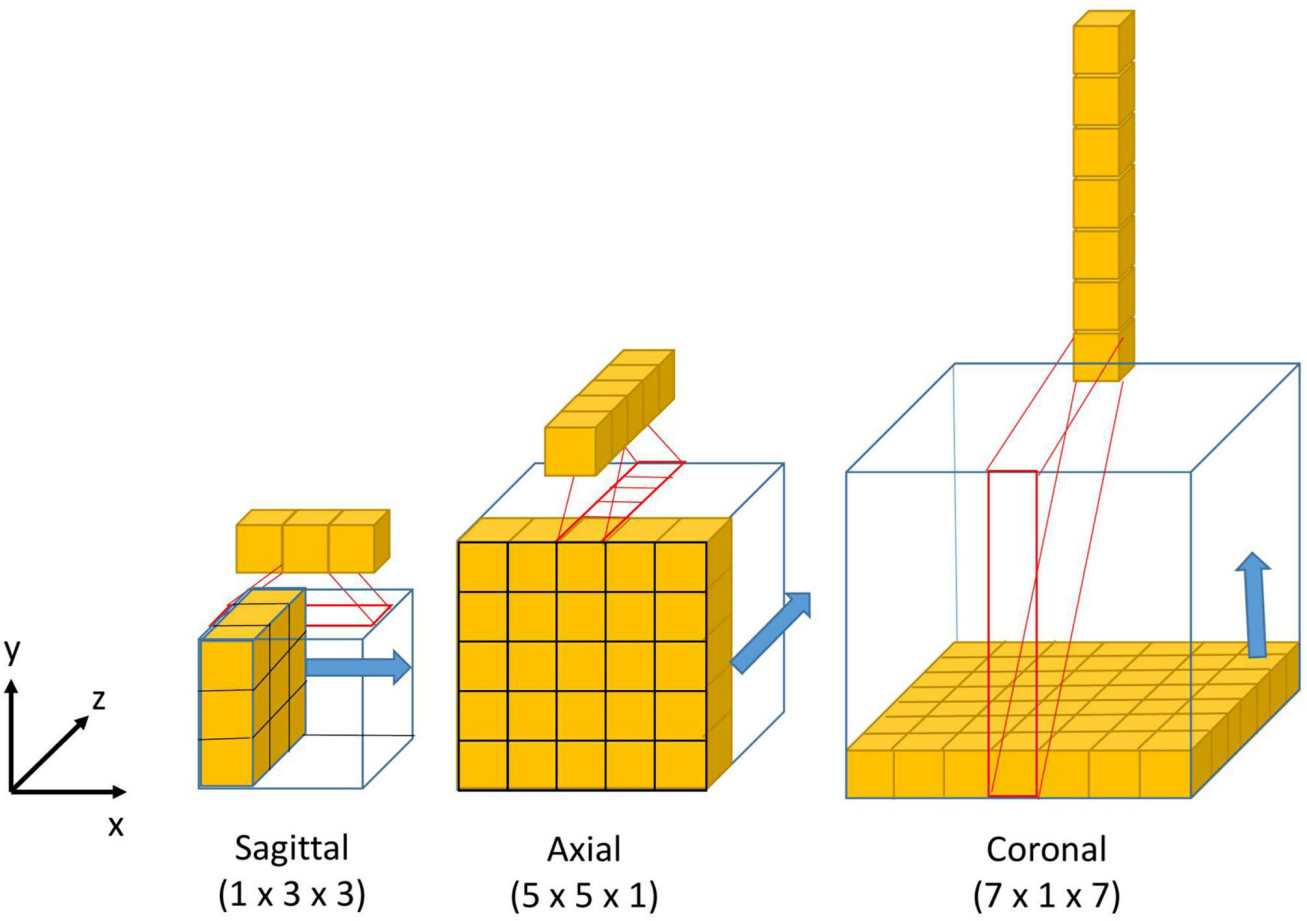
Visual representation of the 3D anisotropic kernels inside each branch of the 2.5D Multi-View Inception block.

The design of the 2.5D Multi-View Inception module is inspired by the Inception module of GoogLeNet (39). Unlike the Inception module, we use 2.5D anisotropic filters instead of 3D or 2D isotropic filters, and we add all the resulted feature maps instead of stacking them. This module has two main characteristics: the first one is the use of convolutions with different receptive field orientation on each image plane—axial, sagittal, and coronal planes—by using anisotropic kernels oriented in each direction. The second is the fusion of the features extracted using anisotropic kernels at different scales. Figure 2 shows the way these two characteristics are combined by replacing a typical 3D isotropic convolution 3 × 3 × 3 into an anisotropic convolution 1 × 3 × 3 followed by a 3 × 1 × 1 convolution. These filters extract features in the sagittal view of the image; the same idea is repeated in “y” and “z” directions but using different scales: 5 × 5 × 1 extracts features in the axial plane and 7 × 1 × 7 in the coronal plane. With this approach, the network extracts and merges features from different planes at the same time, with three parallel branches of anisotropic kernels. After each pair of convolutions, an instance normalization (40) and ReLU (41) activations are applied.

Our model has been optimized using 2.5D convolutions to fit a 12GB GPU memory. 2.5D filters store more weight factors than 2D filters and <3D equivalent filters, with the advantage of obtaining volumetric information on the tumor. Consequently, training an equivalent network with 3D convolutions instead of 2.5D convolutions would require a higher memory capacity (>12 GB GPU). Therefore, our model's design aims to extract volumetric information using 2.5D Multi-view filters and multi-scale information by training a single network. Training a single network instead of three networks, one for each view as implemented in (16, 18) should save training time. Our models take around 8 h to be trained.

#### Asymmetric Ensemble of Asymmetric U-Net (AE AU-Net)

Since 2017 where Kamnitsas et al. (17) won the BraTS challenge using an ensemble of five models with three different architectures, trained with varying functions of loss and using different normalization techniques, all the winners of the following competitions have used ensembles of models as a strategy to improve the segmentation results of their best single models (15, 19–22). Combining different models reduces the influence of hyper-parameter choices and the risk of overfitting, and it is currently the most effective approach for brain tumor segmentation.

In this study, we used an ensemble of models based on the proposed AU-Net architecture and the proposed asymmetric 3D and 2.5D input strategies. This ensemble of models improves segmentation results and the generalization power of our method by reducing the risk of overfitting. Considering the different nature of the input data, we call the proposed method Asymmetric Ensemble of Asymmetric U-Net (AE AU-Net). The proposed ensemble model is based on the training of seven models. According to a 5-fold cross-validation strategy, both the 3D AU-Net and the 2.5D AU-Net were trained five times each with different subsets of the dataset and varying weights of initialization. The seven best-performing models were selected; we finally chose four from 3D AU-Net and three from 2.5D AU-Net, this mainly due to memory constrictions. The ensemble was obtained by averaging the output probability estimates of the labels for each voxel of the image.

### Data and Implementation Details

#### BraTS Dataset

We validated our model in the 2019 BraTS dataset, which consists of pre-operative MRI images of 626 glioma patients. Each patient's MRI images contain four modalities T2-weighted (T2), fluid-attenuated inversion recovery (FLAIR), T1-weighted (T1), and T1 with gadolinium-enhancing contrast (T1c). All images were segmented manually by one to four raters, following the same annotation protocol. Experienced neuro-radiologists approved their annotations. The data are divided into three sets by the organizers of the BraTS challenge: training, validation, and test dataset. The training set is the only one provided with expert manual segmentation and the grading information of the disease. The training dataset contains images of 335 patients, of which 259 are HGG and 76 are LGG. The validation and test datasets include the same MRI modalities for 125 and 166 patients, respectively.

In the training dataset, the ground truth labels are provided for three tumor tissues: enhancing tumor (ET—label 4), the peritumoral edema (ED—label 2), and the necrotic and non-enhancing tumor core (NCR/NET—label 1). From these classes, we defined the following tumor regions to train our models:

- The whole tumor (WT) region. This includes the union of the three tumor tissues ED, ET, and NCR/NET (i.e., label = 1∪ 2 ∪4).
- The tumor core (TC) region. This is the union of the ET and NCR/NET (i.e., label = 1 ∪4).
- The enhancing tumor (ET) (i.e., label = 4).

#### Pre-processing

The multimodal scans in the BraTS challenge were acquired from multiple institutions, employing different clinical protocols, resulting in a non-standardized distribution. The challenge organizers performed several preprocessing steps to homogenize the dataset. The images from different MR modalities were first co-registered to the same anatomical template. The SRI24 multi-channel atlas of a normal adult human brain template (42) was used. The template was obtained by affine registration using the Linear Image Registration Tool (FLIRT) (43) developed by the Oxford Center for Functional MRI of the Brain (FMRIB) and available in the FMRIB Software Library (FSL) (44). The original images were acquired across different views and variable anisotropic resolution. All the images were re-sampled and zero-padded to the same isotropic resolution (1.0 × 1.0 × 1.0 mm) and zero-padded to have the same spatial dimensions (240 × 240 × 155 mm). Skull-stripping was also performed using the Brain Extraction Tool (BET) (45) from the FSL (46).

Even though the images provided were already preprocessed to homogenize the data (4), image intensity variability can still negatively impact the learning phase; contrarily to some other imaging techniques like CT, MRI does not have a standard intensity scale. Therefore, image intensity normalization is often a necessary stage for model convergence. We chose to normalize the MRI images by first dividing each modality by its maximum value and then by centering and reducing the data to have images with the same zero average intensity and unitary SD. This method is widely used due to its simplicity and good qualitative performance (19, 21).

#### Post-processing

We implemented the post-processing of the prediction maps of our proposed model to reduce the number of false positives and enhance tumor detection. A threshold value representing the minimum size of the enhancing tumor region was defined, as suggested in Isensee et al. (19), and the label of all the voxels of the enhancing tumor region was replaced with one of the necrosis regions when the total number of predicted ET voxels was lower than the threshold. The threshold value was estimated as the one that optimizes the overall performance in this region in the validation dataset. Besides, as proposed by McKinley et al. (20), if our model detects no tumor core, we assume that the detected whole tumor region corresponds to the tumor core. We have relabeled the region as a tumor core.

#### Implementation and Training

**Figure 3:**
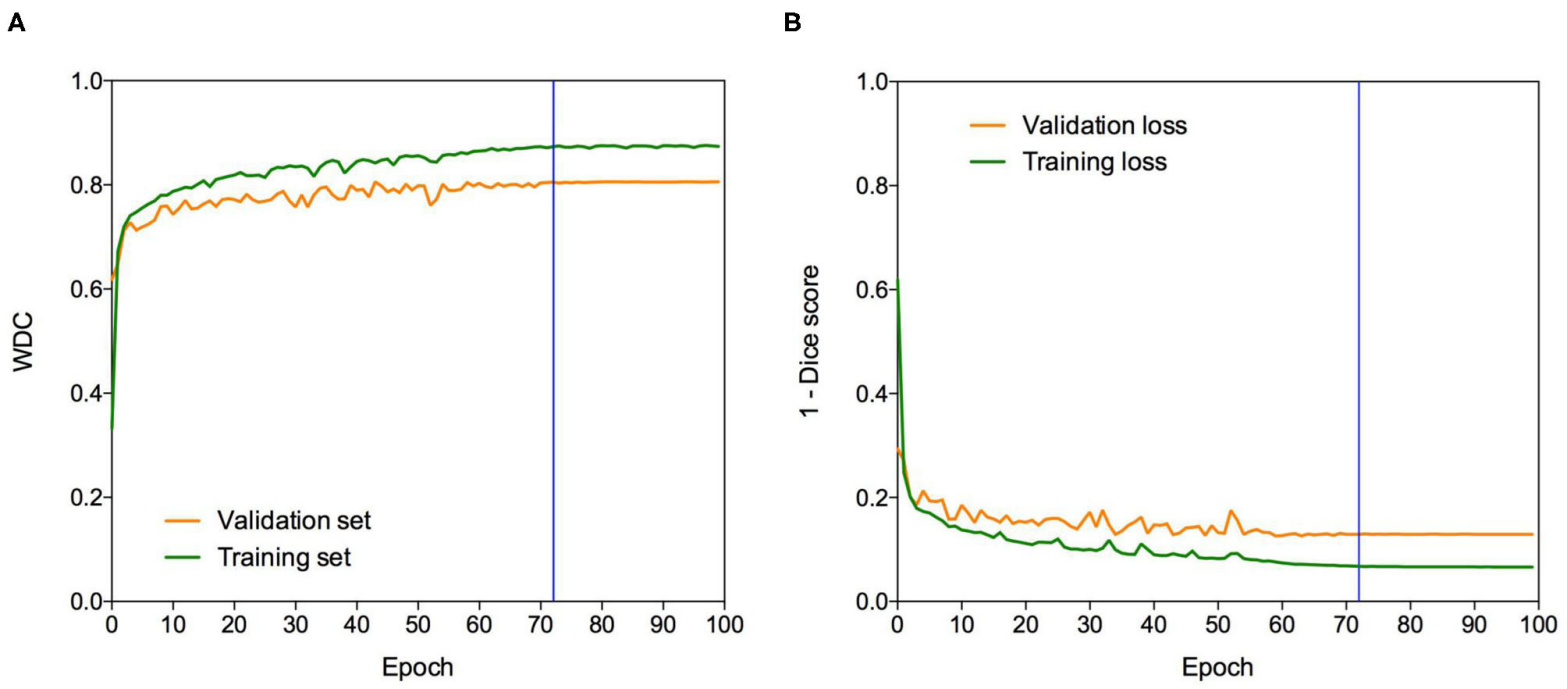
Learning curves. Example of **(A)** WDC evaluation metric and **(B)** (1-Dice score) loss function evolution during training on the BraTS 2019 dataset. The blue vertical line shows the moment at which the model reached the best performance in the validation dataset. The best models were obtained systematically before the 100 epochs.

The Dice loss function was used to cope with class imbalances and weighted Dice coefficient (WDC) as the evaluation metrics to look for the best-performing model (12). Since ground truth segmentations were only provided for the training dataset, we randomly selected 20% from the training set as our internal validation set, taking the same percentage of images from LGG and HGG patients. We trained our models using the remaining 80%. The networks were trained for 100 epochs using patches of 128 × 128 × 128 and Adam optimizer (47) as a back-propagation algorithm, with an initial learning rate of 0.0001 and a batch size of 1. The learning rate was decreased by five if no improvement was seen, on the validation set, within 10 epochs. Our model takes around 8 h to be trained. Figure 3 shows a representative example of the learning curves.

We compared different data augmentation strategies to prevent overfitting, and the best results were obtained using only random crops and random horizontal axis flipping with a probability of 0.5. Note that the data are augmented on the fly during training.

All experiments were conducted on a workstation Intel-i7 2.20 GHz CPU, 48G RAM, and an NVIDIA Titan Xp 12GB GPU. All our models were implemented in Keras (48) 2.2 using the Tensorflow (49) 1.8.0 as a backend. All results obtained on the validation dataset of the BraTS challenge were uploaded on the publicly available evaluation server of BraTS for metrics evaluation. We report the quantitative evaluation values obtained in terms of Dice coefficient and Hausdorff distance, which are defined as follows:


(1) $$Dice\left(P,T\right)=\frac{2\left|P\cap T\right|}{\left|P\right|+\left|T\right|},$$

(2) $$Haus\left(P,T\right)=max\left\{\begin{array}{c} p\in P \end{array}\begin{array}{c} inf\\ t\in T \end{array}d\left(p,t\right),\begin{array}{c} t\in T \end{array}\begin{array}{c} inf\\ p\in P \end{array}d\left(t,p\right)\right\},$$

where *P* and *T* are respectively, the set of predicted voxels and the ground-truth ones, representing the supremum and inf the infimum, and *d* (*p, t*) the Euclidian distance between two points *p* and *t*.

#### Uncertainty Estimation

The use of an ensemble of models improves the segmentation results and the generalization power. Still, it also allows the measurement of epistemic uncertainty and the estimation of structure-wise uncertainty that provides additional information regarding the segmentation results.

To estimate epistemic uncertainty, we averaged the model's output probabilities for each label in each voxel to obtain a new probability measure from the ensemble. Since our model makes a binary classification of each voxel, the highest uncertainty corresponds with a probability of 0.5. Then we used the normalized entropy (Equation 3) to get an uncertainty measure of the prediction for each voxel:


(3) $$H=-\sum_{c\in C}\frac{p_{c}\log\left(p_{c}\right)}{\log\left(\left|C\right|\right)}\in \left[0,1\right],$$

where *p*_*c*_ is the sigmoid output average probability of class *c* and *C* is the set of classes (*C* = {0, 1} in our case). To measure a structure-wise uncertainty, we considered all volumes associated with each region estimated from the ensemble of models. Similar to Wang et al. (16), we calculated the structure-wise uncertainty of each region as the volume variation coefficient (VVC):


(4) $$VVC=\frac{\sigma_{v}}{\mu_{v}},$$

where μ_*v*_ and σ_*v*_ are the mean and SD of the *N* volumes. In this case, *N* is equal to seven. Equations (3) and (4) represent the voxel-wise epistemic uncertainty and structure-wise uncertainty from the ensemble of models, respectively.

#### Uncertainty Measure Evaluation

We evaluate our uncertainty estimation method using the metrics proposed in Mehta et al. (50), which were used in the last two editions of the BraTS sub-challenge on uncertainty quantification to rank the participants. The three-selected metrics aim to reward models when the uncertainty is low in correct predictions and high in the wrong predictions. In this part of the challenge, participants are required to submit along with the brain tumor segmentation, a map of uncertainties associated with each segmentation. The first metric, the Dice area under the curve (Dice AUC), evaluates the Dice score after removing the voxels with uncertainty levels higher than certain thresholds (0.25, 0.5, and 0.75). It is expected that removing uncertain voxels will increase the segmentation accuracy, but it can also decrease the Dice score if many correct predictions are removed.

The two other selected metrics are, respectively, the filter true-positive ratio (FTPR) and the filter true-negative ratio (FTNR), which aim to penalize the elimination of correct predictions, the true positives (TP) and true negatives (TN), respectively. The FTPR is defined as follows:


(5) $$FTPR=\frac{\left(TP_{1.00}-TP_{\tau }\right)}{TP_{1.00}}$$

The FTPR at different thresholds (τ) is measured relative to the unfiltered values (τ = 1.00). The ratio of filtered TN is calculated similarly.

The final uncertainty score for each region is calculated as follows:


(6) $$score=\frac{AUC_{1}+\left(1-AUC_{2}\right)+\left(1-AUC_{3}\right)}{3},$$

which combines the area under the curve of three functions: (1) Dice, (2) FTPR, and (3) FTNR, all as a function of different values of τ.

#### Ablation Study

To evaluate the impact of the proposed AE AU-Net compared with its components, we compared our proposed ensemble model to two variants: (1) 3D AU-Net, which is the AU-Net model with concatenated MRI modalities as the input; (2) 2.5D AU-Net, which is the AU-Net with the proposed 2.5D Multi-View Inception Multi-Input as the input.

## Experiments and Results

### Segmentation Results

#### Qualitative Results

**Figure 4:**
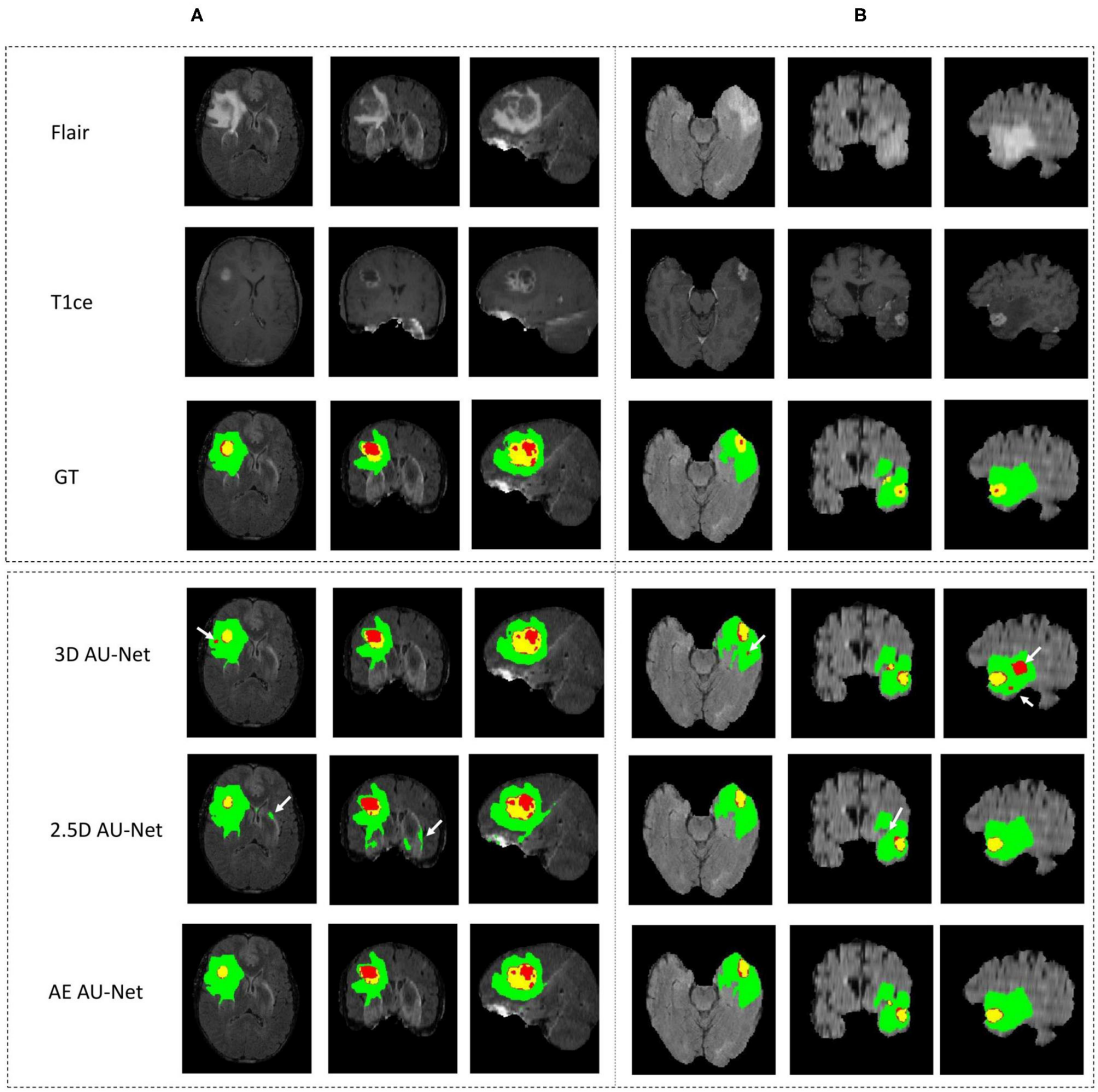
**(A,B)** Qualitative results. Example of segmentation improvement using our proposed model AE AU-Net in two patients from the BraTS 2019 training dataset. The whole tumor (WT) region includes the union of the three tissues (ED, ET, and NET); the tumor core (TC) region is the union of the necrotic and non-enhancing tumor (red) and enhancing tumor (yellow).

Figure 4 shows an example of segmentation results obtained on two HGG patients with 3D AU-Net, 2.5D AU-Net, and AE AU-Net. For simplicity of visualization, only the T1ce and FLAIR images are presented. The three orthogonal views, axial, coronal, and sagittal, are displayed for better representation of the volumetric segmentation. The green, yellow, and red regions correspond, respectively, to the whole tumor (WT), the enhancing tumor (ET), and the tumor core (TC). The ground truth provided with the BraTS dataset is presented on the third row, followed by 3D and 2.5D AU-Net results on rows 4 and 5, and the results of the proposed AE AU-Net are presented in the last row. It can be observed that the segmentation results of the 3D and 2.5D single networks provide proper segmentation, although with some defects that are highlighted with white arrows. Looking at the different views and patients, it can be noticed that the performance of the 3D and 2.5D approaches are varying and that both can present accurate segmentation or defects. In contrast, the proposed AE AU-Net appears globally more accurate, benefiting from both the models to decide more correctly the labeling of the voxels.

**Figure 5:**
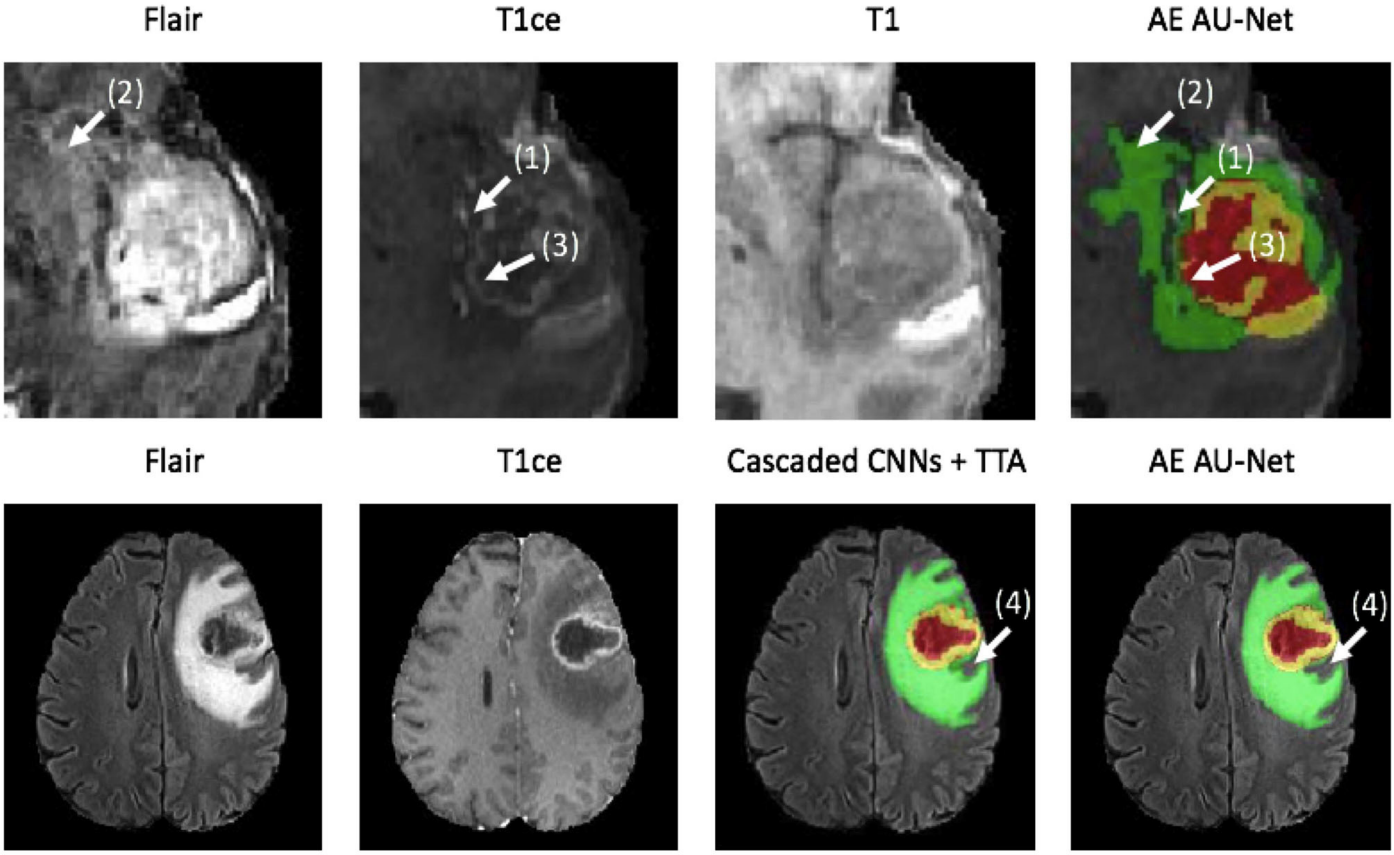
Qualitative results. Two example segmentations using our proposed ensemble. In the top patient identified as CBICA_ZA_1 and in the bottom the patient CBICA_ALZ_1, both from the BraTS 2018 validation dataset. In the bottom, we compare our results with the segmentation made using the Cascaded Networks + TTA presented in (51).

In Figure 5, we present two examples of segmentation made by our AE AU-Net model ensemble. Both patient images were taken from the 2018 validation dataset. In the top, we show the patient identified as CBICA_AZA_1 in the BraTS dataset. We selected the same example reported in Isensee et al. (19) for a qualitative comparison. This case example has been reported as a difficult case for segmentation due to blood vessels close to the enhancing tumor, as can be seen in the T1ce image (arrow 1). Comparing our model segmentation qualitatively with the one reported in Isensee et al. (19), we can say that our model better delimits the region of necrosis and non-enhancing tumor (in green). Our model overlaps better the hyperintense region in the FLAIR sequence (arrow 2) but fails to detect the fine structures of the enhancing tumor region as signaled (arrow 3). Our model effectively excludes the blood vessels (arrow 1) from the tumor, which are small structures with similar image intensity to enhancing tumors. Deep learning models usually fail to detect such small regions due to the few training examples containing these structures. The use of Dice loss function can explain this effect, as missing small structures has a low impact on Dice score and, therefore, a low penalization.

At the bottom of Figure 5, we compare the segmentation made by Wang et al. (51) using cascade networks with test time augmentation (cascaded CNNs + TTA) with the one made by our method for the patient identified as CBICA_ALZ_1. We can see that our model better segments the necrosis and non-enhancing region (arrow 4) but that there are no other significant differences between the segmentations, resulting in similar Dice scores. This suggests that slight differences in Dice values will not always represent significant qualitative differences that could impact clinical practice.

#### Quantitative Results

**Figure 6:**
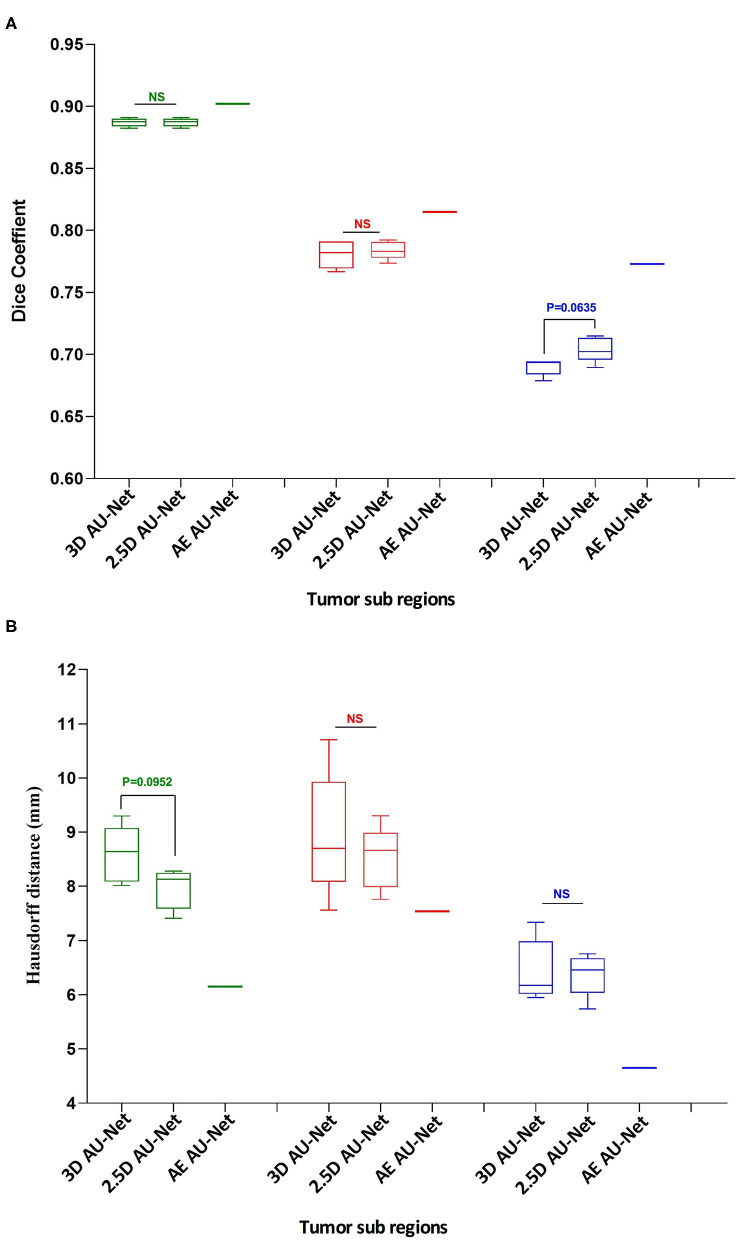
Quantitative results. Box plot comparing two models in a 5-fold cross-validation: the 3D Asymmetric U-Net, the 2.5D Asymmetric U-Net, and the impact of our proposed model (AE AU-Net). **(A)** The *y*-axis presents the Dice score values for the three tumor sub-regions in the *x*-axis. **(B)** The *y*-axis shows the Hausdorff distance for the three tumor sub-regions in the *x*-axis. In green, red, and blue are depicted the three tumor sub-regions: whole tumor, tumor core, and enhancing tumor. The third column corresponding to our AE AU-Net is not a box plot; it is a single value obtained with the ensemble for comparison purposes.

The corresponding quantitative criteria obtained with the different approaches are presented in Table 1. For both the 3D and 2.5D AU-Net, the results correspond to an ensemble from 5-fold cross-validation models. Results for all methods are presented as averagea ± SD obtained over the BraTS 2019 validation dataset. It can be observed that 3D and 2.5D AU-Net got relatively similar scores, with better Dice achieved by 3D AU-Net on the enhancing tumor, and better Hausdorff distance obtained by 2.5D AU-Net on the enhancing tumor and tumor core. The proposed AE AU-Net improved all the scores, reaching the best values on five criteria out of six, illustrating that each architecture learned different features to address the segmentation problem and complement each other. It achieved Dice scores of 0.773, 0.902, and 0.815 on the ET, WT, and TC. For visual comparison, the individual results of the 5-fold cross-validation are presented as box plots in Figure 6, along with *p*-values from Student's *t*-test performed to compare the different models.

**Table 1:** Ablation study on BraTS validation data (125 cases).

		**Dice**			**Hausdorff 95 (mm)**	
	**ET**	**WT**	**TC**	**ET**	**WT**	**TC**
3D AU-Net (5-CV)	0.730 ± 0.284	0.895 ± 0.070	0.796 ± 0.186	6.06 ± 10.54	6.20 ± 9.21	8.40 ± 12.09
2.5D AU-Net (5-CV)	0.714 ± 0.295	0.898 ± 0.066	0.798 ± 0.189	5.74 ± 9.92	6.79 ± 12.6	7.46 ± 9.13
AE AU-Net (5-CV)	**0.773** **±** **0.257**	**0.902** **±** **0.066**	**0.815** **±** **0.161**	**4.65** **±** **8.10**	**6.15** **±** **11.2**	7.54 ± 11.14

*Ensemble results from 5-fold cross-validation are reported for 3D, 2.5D, and AE AU-Net models. The online BraTS evaluation platform computed reported metrics. ET, enhancing tumor; WT, whole tumor; TC, tumor core; 5-CV, ensemble results from 5-fold cross-validation. Bold values highlight the best results for each metric*.

Regarding the Dice metric, no significant difference was observed between 3D and 2.5D models in the WT and TC regions, but a significant difference was observed in the ET region (Figure 6A). Incorporating the 2.5D Multi-View Inception Multi-Input module to the AU-Net improved the result in this region compared with 3D input. The best performance was obtained in all regions with the AE AU-Net, which over-performed 3D and 2.5D AU-Nets with a statistically significant difference (*p* < 0.008). Regarding the Hausdorff distance, it can be observed that the variability of the results was higher compared with the Dice metric (Figure 6B). No statistically significant difference was found between 3D and 2.5D models in the TC and ET regions. In WT, 2.5D AU-Net obtained a significant improvement with respect to the 3D AU-Net model. The smaller Hausdorff distances were obtained in the three regions with the proposed AE AU-Net with a statistically significant difference (*p* < 0.008).

This study has extended our previous work (52), which corresponds to a single model from 2.5D AU-Net architecture. Table 2 shows the results of this single model in the validation and test dataset of the BraTS 2019. We can observe that the 2.5D model achieved a similar overall performance in both validation and test datasets, illustrating that the method did not overfit the validation dataset and generalized well to the test dataset.

**Table 2:** Results of the 2.5D AU-Net model proposed to BraTS 2019 validation data (125 cases) and testing data (166 patients) using our best single model.

			**Dice**			**Hausdorff 95 (mm)**	
	**Best single model**	**ET**	**WT**	**TC**	**ET**	**WT**	**TC**
Validation (125 patients)	2.5D AU-Net (single model)	0.723 ± 0.293	0.888 ± 0.077	0.783 ± 0.206	4.91 ± 8.63	8.12 ± 14.65	7.56 ± 9.40
Test (166 patients)	2.5D AU-Net (single model)	0.775 ± 0.212	0.865 ± 0.133	0.789± 0.266	3.08 ± 3.53	7.42 ± 10.90	6.23 ± 8.50

*The online BraTS evaluation platform computed metrics*.

We have improved the model and the segmentation performance in the validation dataset by implementing the proposed AE AU-Net as an ensemble of seven asymmetric models and using additional post-processing techniques. We also implemented our proposed AE AU-Net framework in BraTS 2018 for comparison purposes. In Table 3, we compared our implementation on BraTS 2018 with a 3D U-Net (53) reimplemented by (51) and cascaded networks (51). We also compare our results with the top-performing methods in the BraTS 2018 challenge, including the first place of the competition (21) and the second place (19). From the comparison with cascade networks, we can observe that our method has a similar performance in terms of Dice in the enhancing tumor and whole tumor regions. We got a comparable although slightly lower performance with the top-performing methods in the same regions, with a lower performance in the tumor core region. We got better performance in terms of Hausdorff distance than cascade networks in the ET and in the WT regions and similar performance with the competition winners.

**Table 3:** Model comparison on BraTS 2018 validation data (66 cases).

		**Dice**			**Hausdorff 95 (mm)**	
	**ET**	**WT**	**TC**	**ET**	**WT**	**TC**
3D U-Net Wang G. et al. (51)	0.734 ± 0.284	0.864 ± 0.146	0.766± 0.230	9.37 ± 22.95	12.00 ± 21.22	10.37 ± 13.47
Cascade networks Wang G. et al. (51)	0.792 ± 0.233	0.903 ± 0.057	0.854 ± 0.142	3.34 ± 4.15	5.38 ± 9.31	6.61 ± 8.55
Cascade networks + TTA + CRF 1	0.803 ± 0.228	0.905 ± 0.054	**0.869** **±** **0.126**	3.01 ± 3.69	5.86 ± 8.16	6.09 ± 7.74
AE AU-Net (our model)	0.800 ± 0.230	0.908 ± 0.055	0.838 ± 0.150	2.59 ± 2.29	4.55 ± 5.92	8.14 ± 13.73
No New-Net Isensee et al. (19)	0.810	0.908	0.854	**2.54**	4.97	7.04
Autoencoder regularization Myronenko (21)	**0.823**	**0.910**	0.867	3.93	**4.52**	**6.85**

*The online BraTS evaluation platform computed metrics. AE AU-Net, Asymmetric Ensemble of Asymmetric U-Net. Bold values highlight the best results for each metric*.

Table 4 shows a comparative performance between the proposed AE AU-Net model and the two best-performing networks presented to BraTS 2019 (15, 22). It can be observed in Table 4 that our method performs closely, although a little less, to the second-ranked submission in the validation dataset and that our model performs better than the second-place model in the enhancing tumor region. The difference in performance in both BraTS datasets, 2018 and 2019, could be explained by the fact that the second performer (19) and (15) did not only use the BraTS dataset to train their model but also an additional Decathlon dataset (54). Regarding the top score (22), it comparatively requires more GPU capacity (>12 GB) to train their model than the proposed method, and Myronenko (21) used 32 GB GPU to train their models. Model comparison in the BraTS tests dataset was not possible since results from the online evaluation platform for this dataset were available only once during the contest.

**Table 4:** Model comparison on BraTS 2019 validation data (125 cases).

		**Dice**			**Hausdorff 95 (mm)**	
	**ET**	**WT**	**TC**	**ET**	**WT**	**TC**
AE AU-Net (our model)	0.773 ± 0.257	0.902 ± 0.066	0.815 ± 0.161	4.65 ± 8.10	6.15 ± 11.2	7.54 ± 11.1 4
2nd Brats competitor Zhao et al. (15)	0.754	**0.910**	0.835	3.84	4.57	5.58
1st Brats competitor Jiang et al. (22)	**0.802**	0.909	**0.865**	**3.15**	**4.26**	**5.44**

*The online BraTS evaluation platform computed metrics. AE AU-Net, Asymmetric Ensemble Asymmetric U-Net. Bold values highlight the best results for each metric*.

### Error and Uncertainty

#### Qualitative Results

**Figure 7:**
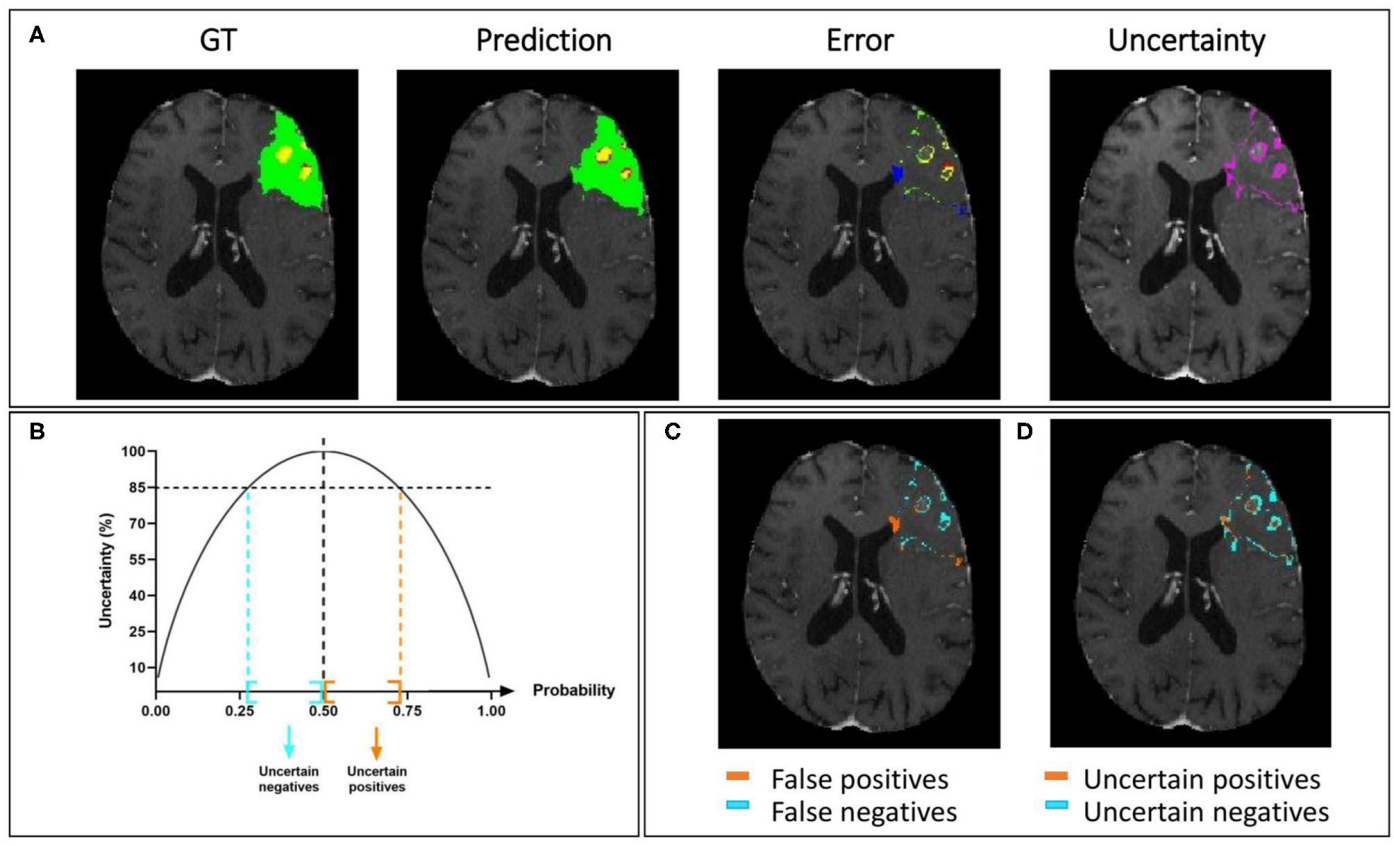
Uncertainty estimation. Example of an HGG patient from BraTS 2019 training set showing **(A)** from left to right: ground truth segmentation (GT), segmentation prediction made by AE AU-Net model, segmentation error, and the estimated uncertainty (threshold 85%). **(B)** Entropy as a function of model output probability showing in blue and orange the intervals for uncertain negative and positive predictions. **(C)** Segmentation error in **(A)** is divided into false positives and false negatives. **(D)** Uncertainty in **(A)** separated in uncertain positives and uncertain negatives. Depicted volumes in green, red, and yellow are whole tumor, tumor core, and enhancing tumor, respectively.

In Figure 7A, we present a representative segmentation result along with the corresponding errors and estimated uncertainty. The errors, which correspond to the difference between the ground truth and the predicted labels, are displayed using the same colors as the ground truth: green, red, and yellow for the whole tumor, tumor core, and enhancing tumor, respectively. The errors in the rest of the brain are displayed in blue. The significative uncertainty, corresponding to uncertainty values above 85 (Figure 7B), is depicted in purple. In Figure 7C, the error is shown with two colors to differentiate false positives and false negatives.

Similarly, in Figure 7D, the uncertainty is displayed with two colors to differentiate uncertain positive and uncertain negative predictions. It can be observed that both the error and the uncertainty were mainly located on the borders of regions, and that their locations are correlated.

#### Quantitative Results

**Figure 8:**
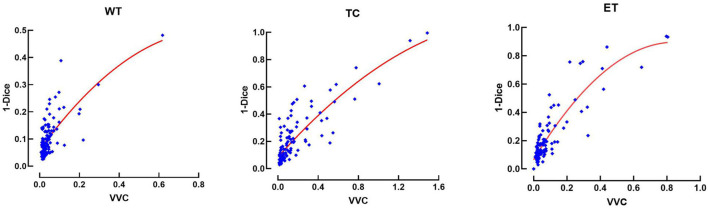
Error (1-Dice) as a function of volume variation coefficient (VVC) for the BraTS 2019 validation set. WT, whole tumor; TC, tumor core; ET, enhancing tumor.

This relationship between errors and uncertainty is further studied in Figure 8, which shows a second-order polynomial regression of the segmentation error (1-Dice) as a function of VVC uncertainty (Equation 4), with an *R*^2^ of 0.49, 0.71, and 0.94 corresponding to the WT, TC, and ET, respectively. It can be observed that the error increases with higher uncertainty. This illustrates that structure-wise uncertainty obtained from prediction variations within an ensemble could be used potentially to train a model further and that it could bring relevant information to the physician. It could also be useful for post-processing to improve segmentation.

We also compare our uncertainty estimation method with other approaches. In **Table 6**, we reported our results in terms of the three metrics used to rank participants in the BraTS sub-challenge on uncertainty estimation, which are described in section 2.2.6. We compare our results with six different methods implemented by Mehta et al. (50). We can observe that our approach performed lower in Dice AUC when filtering out uncertain voxels. However, we perform better both in terms of the FTPR and the FTNR, and our proposed model reached an overall score significantly superior to all the other methods.

#### Single-Input Modalities

Organizing a homogeneous database of MRI images that includes the same modalities for all glioblastoma patients is a challenging task to achieve. In most health centers, the imaging modalities available for each patient may vary, without considering that the image acquisition parameters also change in each protocol. The images in the BraTS database contain the same four imaging modalities for each patient, but in many other databases, they may have only three or even fewer. To evaluate the impact on our model when being trained with only one imaging modality, we trained a single input version of our proposed model with only one of the four modalities separately and also with a combination of two modalities and with all the modalities stacked as a multi-channel input. In Table 4, we compare the results for each modality and a combination of modalities. We observed that using T1, T2, and FLAIR modalities, we obtained the same low value in the ET region of 0.112. This low Dice score is obtained because identifying the enhancing tumor region is only possible with the T1-gadolinium sequence since it requires an enhancement with contrast medium to be identified. The value 0.112 represents implicitly the proportions of tumors that do not have ET at all and for which the BraTS scoring system attributes a score of 1 for correctly finding 0 voxels of this class. For the same reason, there is no Hausdorff distance associated with this region since there were no volumes between which to measure the distance.

The best modalities to segment whole tumor and tumor core were FLAIR and T1-gadolinium, respectively, and the best combination with two modalities was T1-gadolinium and FLAIR. However, the combination of the four modalities was necessary to reach the best possible performance.

#### Multi-View Ensemble

The use of 2.5D convolutions oriented in different directions of the 3D image may pose the dilemma of whether any direction should be pre-selected to segment the tumor better or one of its sub-regions. Wang et al. (51) proposed the fusion of the predictions made by three 2.5D networks oriented in the three different directions of the image (axial, coronal, and sagittal), calling the method Multi-View Fusion. To compare our proposal with the Multi-View Fusion strategy and also to analyze how the orientation of the convolution filters affects the segmentation of each of the tumor's sub-regions, we trained three versions of our model, one for each plane of the image. Each model version with all the filters was oriented in only one of the three orthogonal planes. We applied 3 × 3 × 1, 3 × 1 × 3, and 1 × 3 × 3 convolutions to obtain features in the axial, coronal, and sagittal views. We found that most of the information is extracted from the axial view with better performance in the smaller regions (i.e., the enhancing tumor and the tumor core).

Meanwhile, with sagittal features, better whole tumor segmentation is obtained. The ensemble of the three models from the three different views improves the results in all the regions. The results are shown in Table 5.

**Table 5:** Single input results on BraTS 2019 validation data (125 cases).

		**Dice**			**Hausdorff 95 (mm)**	
	**ET**	**WT**	**TC**	**ET**	**WT**	**TC**
T1	0.112 ± 0.317	0.769 ± 0.178	0.628 ± 0.244	–	12.16 ± 14.52	14.04 ± 16.53
T2	0.112 ± 0.317	0.833 ± 0.138	0.660 ± 0.221	–	9.55 ± 13.34	11.72 ± 10.12
Flair	0.112 ± 0.317	0.865± 0.134	0.656 ± 0.193	–	10.53 ± 20.37	13.23 ± 14.39
T1Gd	0.685 ± 0.322	0.753 ± 0.190	0.749 ± 0.264	**5.16** **±** **6.49**	11.75 ± 11.17	10.34 ± 17.71
T1Gd + Flair	0.674 ± 0.314	0.879 ± 0.091	0.782 ± 0.200	7.43 ± 12.18	8.71 ± 13.96	9.03 ± 12.09
All	0.707 ± 0.302	**0.889** **±** **0.085**	**0.790** **±** **0.202**	5.92 ± 10.41	**7.49** **±** **13.75**	**8.66** **±** **13.03**

*The online evaluation platform computed metrics. The Dice coefficient and Hausdorff distance (mean ± SD) are reported. ET, enhancing tumor; WT, whole tumor; TC, tumor core*.

*Bold values highlight the best results for each metric*.

## Discussion

Most of the proposed methods for medical image segmentation involve the use of architectures that stack all modalities as a multi-channel single input. In previous studies, using multi-input entrance for independent modalities has improved segmenting multiple sclerosis lesions (55) using MRI images, reporting an increase in Dice score metric of around 3%. In the context of the BraTS challenge, this is the first time that the use of multi-input for different image modalities has been evaluated. We designed a multi-input module that aims at extracting features from independent modalities before downsampling the images since, during this process, the specific details from the more informative modalities can be missed by mixing with other modalities. On the contrary, the multi-input approach allows the extraction of more informative modality-specific features for better segmentation. The results that we obtained in the BraTS dataset confirmed our hypothesis. The 2.5D model with multi-input generates more accurate segmentation than the identical 3D model, where all modalities are stacked into a single input. Although the difference in overall performance is slight on average, it was significant in some of the tumor regions, higher by about 2% in the Dice score. This is a non-negligible difference considering that it is difficult to measure improvements due to changes in model architecture (56). However, we found that both the 2.5D and the 3D models predicted relevant information that could be captured in the AE AU-Net that benefits from both. In this study, we found that T1 with gadolinium is the most valuable modality to segment enhancing-tumor regions; the other modalities contribute slightly to the detection of these small regions and when using only the other three modalities independently, our model is not capable to identify this region (see Table 5). Since all the models in the literature that are trained on BraTS datasets use the same four modalities, we believe that their performance is strongly dependent on their availability, in a similar way than our model.

In numerous studies, many strategies have been implemented to combine features of different scales and different views in the image. In this study, we have shown for the first time the use of multi-inputs to combine multi-scale extracted features coming from three orthogonal views of four independent image modalities. While an approach was proposed to combine multi-scale and multi-view features simultaneously (51), it required training separate models for each view. In the proposed 2.5D approach, the three branches in the input module allow the training of a unique 2.5D model. This is convenient in terms of training time since it is unnecessary to train several models with different views or scales, and it can be optimized as a unique process. Besides, using 2.5D convolutions reduces the computational cost. We also compared our implementation with a Multi-View Fusion strategy. Results in Table 6 show the independent assessments of our model trained in different views and the combination of them through an averaging ensemble model. The ensemble improves the accuracy of the segmentation but is inferior to the proposed AE AU-Net approach based on both 3D and 2.5D modules (results in Table 4).

**Table 6:** Different view convolutions comparison on BraTS 2019 validation data (125 cases).

		**Dice**			**Hausdorff 95 (mm)**	
	**ET**	**WT**	**TC**	**ET**	**WT**	**TC**
Axial view (331)	0.671 ± 0.308	0.868 ± 0.106	0.763 ± 0.208	8.81 ± 16.62	11.88 ± 19.45	11.95 ± 17.28
Coronal view (313)	0.651 ± 0.325	0.858 ± 0.119	0.758 ± 0.210	9.46 ± 16.93	14.25 ± 19.26	12.35 ± 18.29
Sagittal view (133)	0.645 ± 0.325	0.874 ± 0.099	0.757 ± 0.208	8.05 ± 14.56	12.96 ± 21.27	11.39 ± 16.67
Ensemble three views	**0.676** **±** **0.318**	**0.886** **±** **0.075**	**0.774** **±** **0.212**	**6.74** **±** **13.19**	**9.08** **±** **15.16**	**9.49** **±** **14.66**

*The online evaluation platform computed metrics. The Dice coefficient and Hausdorff distance (mean ± SD) are reported. ET, enhancing tumor, WT, whole tumor, TC, tumor core*.

*Bold values highlight the best results for each metric*.

When examining the influence of differently oriented 2.5D convolutions in axial, coronal, and sagittal views, results in Table 6 illustrated that in our experiments, the models trained in axial view provide more accurate segmentation in enhancing tumor and tumor core regions. This was predictable since most MRI image acquisitions are obtained with a higher resolution in the axial direction. On the other hand, the models trained in the sagittal view generated a better segmentation of the whole tumor. This is possibly due to the greater volume of the target region, for which the more global information contained in the dimension of lower resolution is more relevant. 2.5D Multi-View Inception modules can be implemented in any architecture and benefit the accuracy of the segmentation. It would also be interesting to assess this block using dilated convolutions instead of using different kernel sizes.

Tables 3, 4 compare our results with the first and second-ranked models in the BraTS 2018 and 2019 challenge. All top-performing methods have in common the use of U-Net like architectures, and all of them used fusing strategies like an ensemble of models.

In the 2018 BraTS challenge, the first-place competitor (21) proposed a variational autoencoder branch for an encoder/decoder model to reconstruct the input image simultaneously with segmentation. The second place used an ensemble of 10 3D U-Net with minor modification models and implemented a co-training strategy using additional labeled images. We also compared our results with the cascade network in addition to test time augmentation (TTA) and a conditional random field for post-processing, implemented by (51). TTA is a data augmentation technique, such as spatial transformations, but applied at test time to obtain *N* different segmentation results combined to get the final prediction.

In terms of optimization in the 2019 BraTS challenge, the first and second place used warming up learning and multi-task learning strategies. The second-place submission of Zhao et al. (15) started from a base model that consists of a U-Net architecture with dense blocks joined with a self-ensemble module that combines predictions made at different scales of U-Net to obtain the final prediction. Additional strategies are then applied to improve performance, including random patch-size training, semi-supervised learning, warming up, and multi-task learning. The authors made an ablation study in which they compared their results using their initial model and after the implementation of three strategies: warming up, fusing, and semi-supervised learning. In our experiments on single models (details not shown for the sake of clarity), we observed that their single initial model has a similar performance to our single model and that our model performs better in enhancing tumor region. However, the strategy that gave them the most significant improvement was the semi-supervised method. The authors used 750 additional cases from Decathlon (54) as the unlabeled data set to implement this strategy.

The best-performing method in the BraTS 2019 competition used a two-stage cascaded U-Net (22). Instead of segmenting one region in each stage in their cascaded strategy, they made a coarse segmentation in the first U-Net like architecture. Then they enter the preliminary prediction map stacked with the original four input modalities into the second U-Net to make the final segmentation. The authors improved their performance implementing a multi-task learning strategy by adding a second decoder to regularize the shared encoder, using the warming up optimization method (57), performing TTA, and implementing a post-processing technique to delete false positives in the enhancing tumor region. The authors obtained similar performances using their single best model compared with fusing 12 models in an ensemble.

Tables 3, 4 show that we got competitive results with top performance methods in the 2018 and 2019 BraTS databases, while training our models with fewer images than Isensee et al. (19) and Zhao et al. (15). We used less GPU memory capacity (12 GB) than Myronenko (21), who trained his models on a 32 GB GPU. We obtained similar performance than cascade networks with better performance in the complete and enhancing tumor regions and with the advantage that our method segments all regions in one step. The cascade networks do it in three stages. First, the whole tumor is segmented. The tumor core is obtained using the previous segmentation to get the segmentation of the smallest region; then, the inference time is also lower in our implementation. In addition, since they used a Multi-View Fusion strategy, they need to train three networks, one for each of three different planes, to make an ensemble of the predictions; in total, nine models should be trained, three for each region. Our implementation requires training seven models.

Our qualitative results comparing top-performing methods with our proposed framework suggest that minor differences in Dice values will not always represent significant qualitative differences that could impact clinical practice.

Comparing the results obtained with the 2.5D AU-Net model in the validation and the test dataset (see Table 2), we are confident that the proposed model generalizes well since similar general performances were obtained. Results obtained in the test set presented an increase in the values obtained in the ET region and a decrease in the WT region. This behavior is also observed in most participating models, making it possible that the dataset test images were easier to segment in the enhancing tumor region and more difficult in the whole tumor region.

Even if we did not observe overfitting behavior, we could improve the performance of our model by adding more complex data augmentation methods, such as elastic and pixel-wise transformations, since some of the top-performing methods in past editions of the BraTS challenge implemented them. However, this was not proposed as there is no clear evidence of the impact of performing more data augmentation (15).

Outside the context of the BraTS challenge, there are some works on brain tumor segmentation. Most of them use hand-crafted features combined with machine learning techniques (45, 58, 59). Soltaninejad et al. (59) used the diffusion tensor imaging (DTI) additionally to the T1, T2, and FLAIR sequences, to improve their method, which combines super voxels and random forest models. While the reported results are comparatively lower than those reported on BraTS challenges, with a Dice value of 0.84 for the whole tumor, they propose innovative modifications that could improve the transferability of trained models.

Deep learning models like the ones trained in BraTS database can be adapted to different types of images, as illustrated by the results of another grand challenge, the Decathlon (54). In this competition, the models must be able to segment a great variety of regions of six different organs, among them the brain, the heart, and the liver, and adapt to other imaging modalities such as CT and MRI. The last winner used an ensemble of three different U-Net architectures, combining 2D and 3D implementations to fit all different image sizes and different memory consumption (19). While being adaptive to many applications, the performance of this framework in the different tasks is usually lower than that of a dedicated end-to-end network.

Finally, we have explored the use of an ensemble of models to estimate structure-wise uncertainties by measuring volume variation coefficients; we observed similar behavior as observed in (51) when using test time augmentation uncertainty estimation, but we found that a quadratic relationship between segmentation error in terms of 1-Dice and structure-wise uncertainty fits better the data (see Figure 8) instead of a linear relationship as reported by (51).

We have compared our method using an ensemble of models to estimate voxel-wise uncertainties; with six different methods implemented by Mehta et al. (50), we found that all the other methods had a higher increase in the Dice score when filtering out uncertain voxels than our method, but this was at the expense of eliminating many true-positive and true-negative predictions. Our method was significantly superior in the final score that considers the average of the three metrics (Table 7). This result suggests that our approach has a better balance between improving Dice coefficient by removing wrong predictions and not drawing too many correct predictions.

**Table 7:** Uncertainty measures comparison on BraTS 2019 validation data (125 cases).

		**WT**		
	**Dice AUC**	**FTPR AUC**	**FTNR AUC**	**Final score**
MC dropout	0.9651	0.1725	0.0175	0.925
Deep ensemble	0.9703	0.2272	0.0160	0.909
Dropout ensemble	0.9711	0.2373	0.0171	0.905
Bootstrap	0.9760	0.2391	0.0179	0.906
Dropout bootstrap	**0.9776**	0.2388	0.0199	0.906
Deterministic	0.9621	0.1697	0.0152	0.926
AE AU-Net (ours)	0.940 ± 0.050	**0.0830** **±** **0.094**	**0.0099** **±** **0.011**	**0.949** **±** **0.05**

*The online BraTS evaluation platform computed metrics. The Dice coefficient area under the curve (Dice AUC), filtered true-positive ratio (FTPR), and filtered true-negative ratio (FTNR) are reported for the whole tumor (WT). Bold values highlight the best results for each metric*.

Our qualitative results in uncertainty estimation show that uncertain voxels are mainly located in the borders of the different tumor regions, which is consistent with other qualitative results in uncertainty estimation (50, 60) and with human-rater variability. We have shown a qualitative relationship between false positives and negatives and uncertain positive and negative predictions (see Figure 7). Quantifying volume variations associated with uncertain positive and negative predictions would be interesting as a future direction to differentiate between possible false-positive and false-negative predictions.

Perhaps one of the most important questions is whether these algorithms have a reliable application in clinical practice. Müller et al., published that CNNs for brain tumor segmentation are not directly applicable in daily clinical practice (61). However, recent works start showing the feasibility of implementing these methods into clinical practice (62, 63). The field continues to evolve and move toward more stable and reproducible methods for different applications such as brain tumor segmentation and stroke detection, where clinical applications are clearly on the horizon (64). Estimating uncertainties will be a crucial factor in achieving these objectives.

As a perspective work, we aim to study the use of uncertainties (both structure-wise and voxel-wise) to improve segmentation accuracy when used as a post-processing technique to filtering out uncertain predictions, taking into consideration the two groups of uncertainties (positives and negatives). Uncertainties could also be used as a reference for user interactions (51) to detect mis-segmentation. We also aim to investigate the reproducibility of this method on images coming from different databases from the clinic.

## Conclusions

We have proposed a novel end-to-end FCN architecture for pre-operative MRI tumor segmentation. We have assessed, for the first time, the use of independent input entrances into an asymmetric U-Net architecture for brain tumor segmentation. Our experimentations illustrated the benefit of the proposed 2.5D Multi-View Inception and Multi-Input module that mixes different views of the 3D images using 2.5D convolutions and the benefit of using it complementarily with a more classical 3D concatenated input in an ensemble of models. The proposed AE AU-Net segmentation aims to balance combining multiple views and different receptive fields and to maintain memory consumption low. We obtained dice scores of 0.902 ± 0.066, 0.815 ± 0.161, and 0.773 ± 0.257 for the complete, core, and enhancing tumor regions, with an overall dice of 0.83 in the BraTS 2019 validation dataset. We also applied our method in BraTS 2018 database with corresponding Dice score values of 0.908 ± 0.055, 0.838 ± 0.150, and 0.800 ± 0.230, respectively. We obtained comparative results with top-performing methods without using any additional training data and requiring less memory to train than the first place in both competitions. In addition, our approach to estimate uncertainties was comparatively superior to other six methods reported in the literature. Finally, the study of voxel-wise and structure-wise uncertainties, and their relationship with the segmentation errors, give perspectives to improve segmentation accuracy further.

## Data Availability Statement

Publicly available datasets were analyzed in this study. This data can be found at: CBICA Image Processing Portal: https://ipp.cbica.upenn.edu/.

## Ethics Statement

Ethical review and approval was not required for the study on human participants in accordance with the local legislation and institutional requirements. Written informed consent was not required to participate in this study in accordance with the national legislation and the institutional requirements.

## Author Contributions

SR-G, TB-S, MH, IZ, and CT conceived and designed the study. SR-G and TB-S contributed to the implementation of the method. SR-G conducted the experiments. SR-G and CT wrote the article. All authors have read and approved the submitted version.

## Funding

SR-G was a recipient of the Ph.D. fellowship number 471721 by the Consejo Nacional de Ciencia y Tecnología (CONACYT) from the Mexican Government.

## Conflict of Interest

The authors declare that the research was conducted in the absence of any commercial or financial relationships that could be construed as a potential conflict of interest.

## Publisher's Note

All claims expressed in this article are solely those of the authors and do not necessarily represent those of their affiliated organizations, or those of the publisher, the editors and the reviewers. Any product that may be evaluated in this article, or claim that may be made by its manufacturer, is not guaranteed or endorsed by the publisher.
